# Exploring Medical Student Attitudes Regarding Inter-university Learning: A Pilot Study

**DOI:** 10.7759/cureus.63260

**Published:** 2024-06-27

**Authors:** Kate Ridley, Elinor Jones, Courtney Johnson, Emily Collman, Chris Jacobs

**Affiliations:** 1 Postgraduate Medical Education, Great Western Hospitals NHS Foundation Trust, Swindon, GBR

**Keywords:** preparedness for practice, novel approaches to education, simulation medicine, working together, inter-professional practice, inter-university learning, medical school education, simulation in medical education

## Abstract

Introduction

The United Kingdom needs to educate more medical students to meet workforce demands. With static numbers of clinical teachers available, novel and efficient approaches are required to prepare students for real-life work where doctors routinely work with colleagues from different medical schools. This innovative project was designed to investigate student attitudes towards inter-university learning (IUL), whereby two medical students from different universities learn together.

Materials and methods

Thirteen students at Great Western Hospital, Swindon, England, volunteered and were randomly paired with a student from another university. Pairs completed a 20-minute simulated clinical scenario and observed three others. Students completed pre- and post-session questionnaires adapted from the Readiness for Inter-Professional Learning scale. Seven students took part in semi-structured interviews which underwent thematic analysis.

Results

Quantitative analysis of post-session questionnaires demonstrated a positive response to IUL. Thematic analysis generated six themes: impact on learning, impact on career, working together, recognising differences, practical considerations, and psychosocial perspectives.

Discussion

Students enjoyed the social learning opportunity to practise team-working, communication, and role delegation with unknown peers whilst sharing different clinical approaches. Differences in course structure meant students displayed varying strengths, although unexpected findings centred around pre-conceptions of both universities and social comparison behaviours.

Conclusion

IUL’s strength was deemed to be in non-technical skill development to prepare for real-life work, ultimately enhancing patient safety. Practicalities to consider include session design and psychological safety. IUL provides a novel solution to efficiently educate future healthcare professionals and further work to explore its benefits on a wider scale is suggested.

## Introduction

The Medical Schools Council (MSC) in the United Kingdom (UK) has recommended that 5,000 extra medical student places per year are needed to support a sustainable workforce. It has recognised that clinical placement capacity poses a challenge in supporting the expansion of medical schools and that novel approaches to teaching are needed if this expansion is to be feasible [1]. This, paired with static numbers of clinical teachers, presents obstacles that need to be overcome to maintain high-quality medical education.

Whilst traditionally a significant portion of clinical education is done by National Health Service (NHS) staff, the workforce is currently facing the worst crisis in its history, and physician burnout, reaching record levels globally, is driving career disengagement [2,3]. The authors are concerned about the impact that this may have on teaching, which the General Medical Council (GMC) cites as a core role of doctors [4]. These factors, combined with expansion barriers cited by the MSC, stress that new approaches are needed to facilitate larger numbers of students on hospital sites, where staff with expertise and willingness to teach can be used in ways that make efficient use of their time. The MSC recognises that medical schools need to develop ways to accommodate the education of more students than they have previously [1].

Further to this, it is recognised that a shift in preparing healthcare students for clinical work is needed, particularly in the areas of acute care and non-technical skills [5]. The transition from medical student to junior doctor is significant, and easing this shift should be seen as a priority for medical schools. Challenges such as navigating colleague personalities, teamwork, and role identification are commonly reported [6].

Inter-professional education (IPE) is endorsed by the World Health Organisation for its value in preparing healthcare students for collaborative practice and teamwork [7]. The practice is now mandated by the GMC and is thought to improve patient care [4]. Commonly, IPE is delivered in the form of simulation which allows students to practise skills without consequence to promote collaborative relationships [8]. A review of IPE learning methods reported that authenticity is crucial to the experience, which can be facilitated through simulation, and whilst classroom-based IPE can teach about teamwork, it does not allow students to experience it [9,10].

Recognising the benefits of IPE, the authors sought to adjust interprofessional groups to those of medical students from different universities and design a research study on inter-university learning (IUL) whereby medical students are offered the opportunity to learn with students from different medical schools. Currently, a gap exists in traditional teaching methods where educational interaction between medical students from different universities is lacking. When graduating from a medical school in the UK, doctors initially become Foundation Year One (FY1) doctors. These FY1 doctors are expected to work effectively with graduates from different medical schools, yet opportunities to participate in a community of practice are not offered prior to graduation.

Whilst doctors should be able to work with colleagues from any medical school, it is actually clear that universities adopt different teaching styles and approaches; this means that medical students are ultimately different on graduation [11]. Without an understanding of their commonalities and differences, how can medical students be expected to work well together with no practise? Lave et al. proposed that learning is an “integral and inseparable aspect of social practice” [12]. IUL provides an opportunity for students to engage in social learning, allowing them to explore their differences and find their commonalities whilst also preparing them for real-life work. Current literature on IUL is limited to community-based clinical attachments, reports of inter-professional IUL, and online learning environments during the pandemic [13-15]. It is unclear whether IUL has been explored in undergraduate medical education within the UK.

Researchers hypothesised that the potential benefits of IUL might include improving students' preparedness for practice by replicating real-life work. Prior to exploring this initiative on a wider scale, researchers designed this study to investigate student attitudes towards IUL, understanding that, for IUL to be successful, it must also be accepted by students. The existing literature on the success of simulation use in closely related IPE encouraged the authors to adopt simulation as the teaching modality for this study. The objective of this study was to quantify what medical students felt about the concept of IUL prior to experiencing this teaching method and how, if at all, those attitudes were changed following exposure to this learning style.

This article was previously presented as an oral presentation at the 2022 Association for the Study of Medical Education Annual Scholarship Meeting.

## Materials and methods

In January 2022, 13 fourth-year medical students at Great Western Hospital (GWH), Swindon, England, enrolled voluntarily in this study. Eight students were recruited from the University of Oxford, and five students were recruited from the University of Bristol. Students were recruited via email advertisements and word of mouth, with the following inclusion criteria: medical students from the University of Oxford or Bristol in their fourth year of study AND previous simulation experience either at GWH or another centre. It is worth noting that Oxford students are taught with a traditional approach, separating preclinical and clinical education, whereas Bristol students are taught using case-based learning (CBL) and experience earlier clinical contact. This means that the students were not equally matched in terms of clinical experience; however, scenarios were chosen by facilitators who had knowledge of both universities’ curricula, and the scenarios were considered to be at the appropriate level for all students. Scenarios included asthma, sepsis, pulmonary oedema, and gastrointestinal bleeding. These were felt to be academically stimulating but not overwhelming, allowing the students to focus on teamwork and non-technical skills.

Students attended one evening session in the hospital’s simulation suite. They were randomly allocated a unique number as their identifier and randomly paired with a student from the other university. Each pair completed one 20-minute clinical scenario and observed three other pairs in a separate room via video link. The discrepancy between the number of Oxford versus Bristol students was contributed to by unexpected absences. Given that students were still keen to participate in the session, there was one occasion where two Oxford students worked together and another where one Bristol student participated twice. Whilst we recognise that this was not the intended structure of the session, IUL still occurred between students during the pre-brief and debrief sections.

Students received a pre-brief highlighting that the simulation was a safe learning environment where they could ask for help or stop the scenario at any time. Observing students were encouraged to discuss the case, and integration was promoted by alternating the seating arrangements between Oxford and Bristol students. After each case, participants regrouped and discussed the case, facilitated by a member of the research team who was clinical teaching doctors. In an attempt to avoid bias in students' post-session responses, the discussion was primarily focused on the medical aspect of the case; however, naturally, some elements of teamwork and communication skills were discussed.

Written pre- and post-session questionnaires, adapted from the Readiness for Inter-professional Learning (RIPL) scale, were used with 5-point Likert scale responses [16]. Students completed these immediately before the session began and after. Data were treated as continuous, and a paired t-test was analysed on pre- and post-simulation IUL survey scores using Stats Direct (version 3.3.5), with negative questions given a reverse score.

Students were asked to volunteer to attend a one-on-one semi-structured interview with a researcher (Table 1). Seven students (four Oxford, three Bristol) agreed to attend. These interviews were completed within 72 hours, with the intention of minimising recall bias. The interviews were recorded and transcribed using Microsoft Teams software, and this was manually cross-checked. Four researchers completed an inductive thematic analysis using Braun et al.'s six steps to thematic analysis over a three-month period [17]. It was felt that over 50% of the participants would be a reasonable representative sample size for a small pilot study. By focusing on a smaller number of interviews, researchers felt there would be sufficient time to conduct high-quality thematic analysis. Ethical approval was granted by the Swindon Academy Medical Education Research Committee.

**Table 1:** Semi-structured interview questions written by the research team

Semi-Structured Interview Questions	
1	What experience of inter-university learning did you have prior to taking part in this project?
2	How do you feel about working with a medical student from another university?
3	How did you feel the simulation scenario went?
4	What are your reflections on how you worked as a team with the other student?
5	How do you think that this experience might impact you when you first start working as a junior doctor?
6	What are the benefits of inter-university learning?
7	What are the disadvantages of inter-university learning?
8	What do you think the benefits or disadvantages are of IUL for patient safety and patient care?
9	What are the barriers to inter-university learning?
10	Have you got any other comments or opinions that you'd like to share about inter-university learning?

## Results

Questionnaires were completed by 100% of participants. All participants were 19-23 years old, five of which were females and seven males. No further demographic data were collected. Seven interviews were attended (three females and four males).

Quantitative data analysis

**Figure 1:**
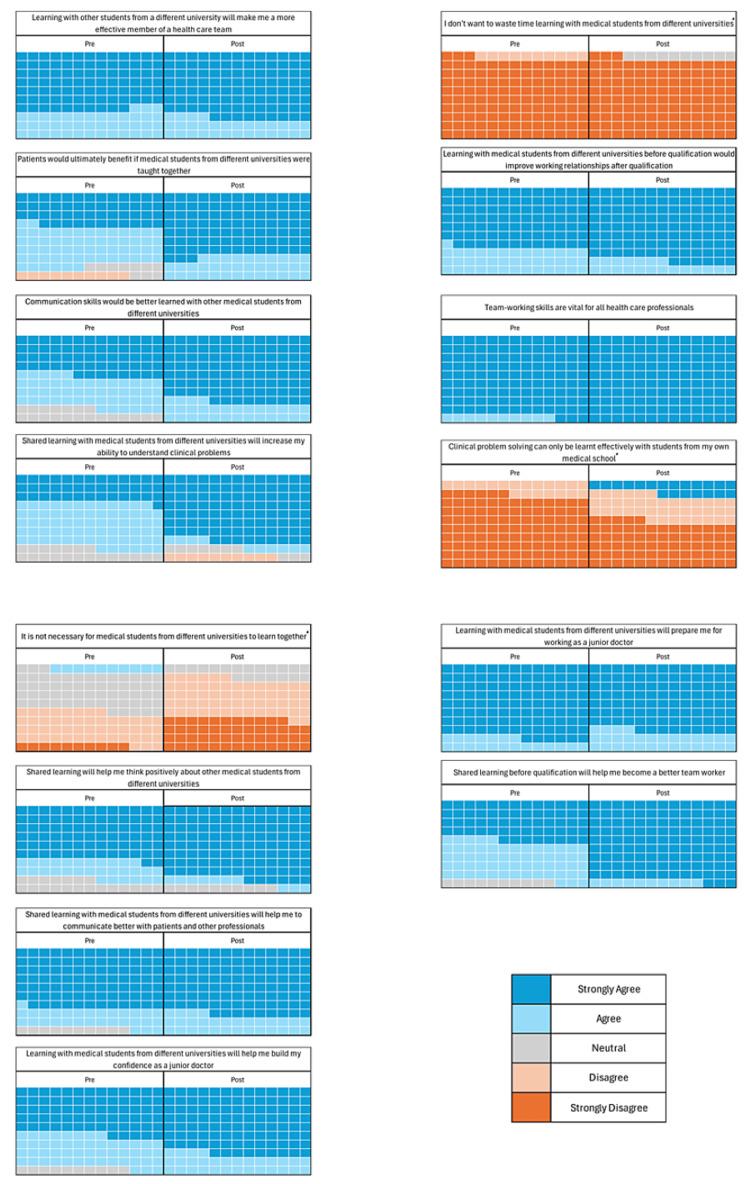
Waffle charts of pre- and post-questionnaire responses Key included. Data from 13 participants showed a positive trend in opinions after completing the inter-university simulation. (10 squares represent one participant). Questions with * indicate reverse marked responses.

IUL total questionnaire scores (maximum score of 70) were compared before and after the simulation. On average, the total scores after the IUL simulation (mean (M)=65.85, standard deviation (SD)=4.79) were higher than before the simulation (M=62.85, SD=4.31) (Figure 1; negative questions are marked with an asterisk). This improvement (3.00, 95%CI (-4.86, -1.13)) was statistically significant (t(13)=-3.51, P<0.05).

Thematic analysis

 Six themes were identified through thematic analysis, with 18 sub-themes (Table 2).

**Table 2:** Themes and sub-themes identified from the thematic analysis of interviews.

Psychosocial perspectives	Practical considerations	Impact on career	Impact on learning	Working together	Recognising differences in medical schools
Apprehension	Simulation design	Teamwork	Understanding theory versus non-technical skills	Trust	Course structure
Representation	Logistics	Improving patient care	Learning new approaches	Roles and delegation	Perceptions of the students from their own university
Self-criticality	Student matching	Preparedness for work	Enjoyable learning experience	Teamwork and communication within the scenario	Perception of students from other university

## Discussion

Impact on learning 

Students positively reflected that IUL provided learning opportunities through exposure to the other university students’ approach to the same clinical problem: "I really found it helpful.. maybe they learned from me, but I definitely learned a lot from them" (Interviewee 6). Students felt they learnt these new approaches even when allocated observer roles, as supported by Bandura’s observational learning theory, cited by Bethard's and further facilitated by scenario debrief discussions [18]. In line with peer-assisted learning theory, students enjoyed sharing knowledge, but IUL also highlighted gaps, which motivated them to improve [19]. Interviewee 1 stated that "I think having the sort of contact with other universities definitely is a benefit 'cause you sort of see where you're lacking out and where you can improve on when you're training."

Students reflected that IUL develops non-technical and interpersonal skills, such as communication and teamwork. When asked what the benefits of IUL are one student replied "I guess when you suddenly have to work together then that's a skill in itself. It’s good to navigate new roles with people you don’t know. So I think that's the main thing" (Interviewee 3). These crucial qualities are outlined in the GMC’s Outcomes for Graduates [20]. Meanwhile, students recognised that IUL may be less beneficial for those focusing on university-specific exams: "I think it really depends on what you're learning... if you are trying to learn the pathology... It might actually be quite nice to do it with your own uni... at least for the sake of my finals" (Interviewee 1). Some felt that a similar experience could be gained through same-university learning with unknown peers. Overall, after participating, students tended to agree more (85%-100%) that non-technical skills are better learned with students from other universities. Following the session, two students did change their response from agree to strongly disagree when asked “Clinical problem solving can only be learnt from my own medical school”, and researchers postulate as to whether this is because there are certain aspects of the curriculum that students prefer to learn with their own cohort.

Importantly, students enjoyed the experience and reflected on the positive social aspects of IUL: “We all went back and told the others that it was actually really worthwhile and it was good not only for your own revision, but just a nice almost social experience as well” (Interviewee 3).

Impact on career

Preparedness for working is influenced by personality traits, course design, and perceived relevance of course content [21]. Students reflected that learning to work effectively with strangers was relevant to prepare them for real-life work: “when we begin as F1s we’re [going to] have to work with everyone regardless… it's nice to have already [gotten] that experience now” (Interviewee 1). Facilitating sessions that are seen as relevant to students’ upcoming social roles is a key feature of adult learning theory and drives engagement [22]. Students involved in this study were fourth-year students and appreciated the need for this training; an interesting area of further study would be to include final-year students who are even closer to graduation.

This study highlighted that medical students from different universities demonstrate different strengths and weaknesses. Students discussed that the leadership role was always allocated to Bristol students who were deemed to have greater clinical experience. Research has shown that doctors with less clinical experience often find it difficult to take the lead in emergency situations and that less experienced doctors tend to be more “hands-on” [23]. Students felt that this was representative of real-life medical teams and concluded that this variation of skills could benefit patient care.

Lastly, students discussed social anxiety associated with starting foundation training and felt that meeting and working with other medical students would prepare them for this - “it's good practice to get used to meeting new people and working with them straight away” (Interviewee 2).

Working together 

Learning teamwork skills before qualification is essential as poor communication can risk patient safety [24]. Without IUL, medical students may be ill-equipped for real-life work with other doctors. Salas et al. proposed "The Big Five" model of teamwork, which highlights the importance of communication and mutual trust in a team, particularly in emergencies [25]. Students reflected that, when working with unknown peers, direct verbal communication was more frequently needed compared to relying on non-verbal cues when working with familiar peers. Having completed IUL 92% of students strongly agreed that learning with others from different universities would make them a better team worker, with only 46% strongly agreeing prior to the experience.

"The Big Five" model also illustrates the need for mutual trust in a team, which was challenging for our students. Students did not know what each other had covered in medical school and, as a result, were unsure of each other’s competencies. Students felt that they had to openly voice their strengths and weaknesses in IUL when compared to working with a student from the same university: "I think it's quite an important skill in medicine to say I don't feel comfortable doing this safely and handing it over to someone more senior with more experience to do it" (Interviewee 4). Aware of being observed, some students did not want to come across as “inferior” to the other student, whereas others were cautious of being “overbearing”, but ultimately reflected that voicing their clinical ability was essential: "I struggled in that because it was all about everyone watching me and then I didn't want to be like over powering of the person, but I didn't want to not say anything when they were needing help" (Interviewee 7).

Recognising the differences in medical schools 

Students were acutely aware of the differences between their medical school course structures and the associated approaches to learning. Differences in medical schools' teaching styles have been well-studied by the AToMS study [11]. The impact of these differences has been studied by the high-powered MedDifs study, which concluded that more measures need to be in place to effectively evaluate the impact of different medical schools on their graduates [26]. It is important to note that skills such as communication, teamwork, and leadership cannot be easily quantified and therefore compared between schools.

Accepting that there are differences is crucial, and our students reflected that IUL was an opportunity to increase their awareness of these differences. The GMC stated in 2014 that “variation between medical schools in the interests, abilities and career progression of their graduates is inevitable and not in itself a cause for concern” [27]. However, a movement towards a national standard examination in the UK suggests that there is an aim for more uniformity across medical schools. IUL allows undergraduate students an opportunity to learn together and find their commonalities, with one student commenting that “it's not as if, like, we're doing a completely different alphabet, like we've learned the same A-E assessment” (Interviewee 2) whilst also exploring how they were different. This will help students as they become doctors to navigate their careers and working relationships.

We did not anticipate that students would discuss pre-conceptions of students from their own and the other university at such length. It was perceived that Oxford students had greater knowledge, whilst Bristol students had superior communication skills due to differences in clinical exposure and their course structure. These preconceived ideas translated into assumptions about the personal characteristics and clinical competencies of the students. Whilst some students felt IUL risked reinforcing negative stereotypes - “...we had stereotypes about the other university, so I guess like the session was either going to prove them, or like disprove them…” (Interviewee 2) - most felt the sessions would allow for the debunking of these: “...it's nice to, like, kind of get rid of that [the preconceived ideas] before you go on wards and get to know people in a work setting” (Interviewee 6); “in reality, you know, we're kind of in the same boat” (Interviewee 5). Awareness of these issues within IUL is important, and further exploratory work is suggested.

Practical considerations 

When adopting IUL, readers will need to consider practicalities. Students were conflicted on whether they would travel for IUL, highlighting possible limitations in the practice if students are not allocated to common placement sites. Other logistics such as the start time of the session should be considered; this project was run as an additional optional evening session due to time constraints on both students and teachers, which may be a less favourable approach amongst both parties in the long term. We recognise that, in its infancy, IUL may be introduced as an optional addition to the undergraduate curriculum, but we hope that, in time, IUL will become integrated into standard teaching programmes. Cohort size will need to be considered when addressing the feasibility of implementing IUL amongst all students.

As well as logistics, IUL facilitators will need to carefully consider session content and delivery to ensure student psychological safety. Ensuring students that feel safe to learn is a longstanding concept in medical education [28]. In this study, students who participated were all in the fourth year of their medical studies. Students reported that the same year groups are not comparable across universities and increased levels of anxiety were attributed to being paired with someone with greater levels of clinical experience. Interviewee 6 stated that "I was nervous because I knew that they had more clinical skills than we did, so I felt like I was going to hinder my partner a little bit." This may pose challenges when designing sessions appropriate for the level of all students. Educators are encouraged to consider this when matching students from different universities. Having an understanding of topics already covered by both universities and choosing content that incorporates this gives students the capacity to build upon their non-technical skills and teamwork, rather than focusing on new medical knowledge. 

Psychosocial perspectives

Students initially felt apprehensive prior to IUL, sometimes due to feeling inferior to the other university’s students, which was often due to perceived differences in knowledge and clinical exposure - “(the other students) have probably done this kind of stuff before and will kind of know how to react and respond in these situations, which we probably won't” (Interviewee 5). Whilst there may be a correlation between clinical experience and self-rated confidence amongst medical students, neither of these in greater measure necessarily correlates with better performance in simulated or written assessments [29]. Despite this, the sub-theme of self-criticality ran throughout the transcript data. Students expressed fears of judgement from others; they felt self-conscious of how they were being perceived and were comparing themselves and their abilities to that of others: "I assume they might think oh, we are very nerdy or..might even think we do know what we're doing" (Interviewee 5). Some students felt pressure to perform well "you wanted to show off your best ability" (Interviewee 3) and represent their university. Meanwhile, others felt less pressure due to greater anonymity - “In this instance it's kind of nicer working with people you don't know 'cause you're never gonna see them again... if it goes wrong, that's fine. They don't know me. And then you move on…” (Interviewee 6).

Social comparison, where the experience and perception of peers are used to estimate one’s own ability to succeed, is known to contribute to self-confidence and perceived self-efficacy. Given that social comparison can distress students and impact their learning, it is imperative that those adopting IUL are mindful of this [30]. Some participants highlighted that IUL was an opportunity to build self-confidence, which may reflect the fact that students can display differing comparison behaviours. Some students have a greater tendency to compare themselves to others or may be more affected by these comparisons than their peers. Additionally, comparisons may be motivated through seeking self-enhancement or self-improvement. This depends on whether peers are more likely to be seen as inferior or superior to the individual [30]. If educators are concerned about the impact of social comparison amongst students participating in IUL, it may be helpful to encourage students to reflect on their motivations and orientation tendencies when they compare themselves to others [30].

Limitations

This pilot study was limited by its small sample size. Restricted access to simulation suite resources and researcher work schedules meant that it was only feasible to recruit 16 students. As the IUL sessions had to be offered out-of-hours due to the availability of the simulation suite, it is therefore possible that other personal commitments may have impacted the ability of students to sign up or travel to the sessions. Hence, only 13 students were recruited rather than the target of 16.

The ratio of Oxford-Bristol students recruited was unequal. In addition to the aforementioned unexpected absences, this may be due to the timing of the simulation sessions in relation to the University of Bristol's scheduled holidays; whilst sessions were planned within formal term dates, some students may have opted to leave their placement early.

The above limitations may have been mitigated by offering more sessions and carrying out the project over a longer time period, but this was not possible within the scope of this pilot study.

## Conclusions

With medical school spaces expanding and the NHS facing a workforce crisis, novel approaches are needed to efficiently educate future healthcare professionals. IUL provides an opportunity for clinical educators to teach students from different universities simultaneously, and whilst not explored in this small pilot study, the authors anticipate that the practice will reduce the burden on teachers, and this could be an area for further research. In order for IUL to be successful, it has to be acceptable to students, and this study was designed to ascertain what student's attitudes are towards IUL. Overall, the results of our study obtained through pre-questionnaires suggested that students believed the concept of learning from students from other universities is valuable and positive. Following their experience with IUL, the improvement in their post-questionnaire scores favouring IUL was statistically significant.

Thematic analysis allowed us to further understand the students’ attitudes to IUL. As well as finding it enjoyable, students feel that IUL is useful for learning different ways to address clinical problems and has relevance in preparing them for real-life work. IUL provides an opportunity for students to explore their commonalities and differences within their community of practice. Key strengths of IUL, are not limited to, but include the development of communication and teamwork skills. When implementing IUL in their own institutions, facilitators must be mindful of social comparison behaviours amongst students and other practical considerations of session design, student matching and logistics. Researchers appreciate the sample size of our pilot was small and further work to explore the benefits and disadvantages of IUL on a wider scale is suggested. However, this study highlights an exciting teaching modality that is acceptable to students, and we are excited to see what role this unique practice will play in the future of clinical education.
